# Survey of *Bartonella* spp. in U.S. Bed Bugs Detects *Burkholderia multivorans* but Not *Bartonella*

**DOI:** 10.1371/journal.pone.0073661

**Published:** 2013-09-09

**Authors:** Virna L. Saenz, Ricardo G. Maggi, Edward B. Breitschwerdt, Jung Kim, Edward L. Vargo, Coby Schal

**Affiliations:** 1 Department of Entomology and W. M. Keck Center for Behavioral Biology, North Carolina State University, Raleigh, North Carolina, United States of America; 2 Intracellular Pathogens Research Laboratory, Center for Translational Medicine, North Carolina State University, Raleigh, North Carolina, United States of America; 3 Structural Pest Control and Pesticide Division, North Carolina Department of Agriculture and Consumer Services, Raleigh, North Carolina, United States of America; U. Kentucky, United States of America

## Abstract

Bed bugs (*Cimex lectularius* L.) have resurged in the United States and globally. Bed bugs are hematophagous ectoparasites of humans and other animals, including domestic pets, chickens, and bats, and their blood feeding habits contribute to their potential as disease vectors. Several species of *Bartonella* are re-emergent bacterial pathogens that also affect humans, domestic pets, bats and a number of other wildlife species. Because reports of both bed bugs and *Bartonella* have been increasing in the U.S., and because their host ranges can overlap, we investigated whether the resurgences of these medically important pathogens and their potential vector might be linked, by screening for *Bartonella* spp. in bed bugs collected from geographic areas where these pathogens are prevalent and from bed bugs that have been in culture in the laboratory for several years. We screened a total of 331 bed bugs: 316 bed bugs from 36 unique collections in 29 geographic locations in 13 states, 10 bed bugs from two colonies maintained in the laboratory for 3 yr, and 5 bed bugs from a colony that has been in culture since before the recent resurgence of bed bugs. *Bartonella* spp. DNA was screened using a polymerase chain reaction assay targeting the 16S–23S rRNA intergenic transcribed spacer region. *Bartonella* DNA was not amplified from any bed bug, but five bed bugs from four different apartments of an elderly housing building in North Carolina contained DNA sequences that corresponded to *Burkholderia multivorans*, an important pathogen in nosocomial infections that was not previously linked to an arthropod vector.

## Introduction

Bed bugs (*Cimex lectularius* L.) are an important resurging pest in urban centers globally, including the U.S. [1]. These hematophagous ectoparasites feed mainly on humans but they also occasionally feed on other animals including bats, chickens, and domestic pets such as cats [2], [3]. Because of their blood-feeding and commensal association with their hosts, there is great concern about the potential of bed bugs to vector disease organisms. More than 45 potential human pathogens have been isolated in association with bed bugs, but none has been shown to be transmitted from bed bugs to humans (review: [4]). Furthermore, experimental infection of bed bugs with pathogens in the laboratory showed that hepatitis B virus could persist in the insects and their feces for up to 5 wk [5], and the spotted fever group rickettsia, *Rickettsia parkeri*, could last up to 2 wk in the insects [6].

*Bartonella* is a genus of emerging and re-emerging facultative intracellular bacterial pathogens found throughout much of the industrialized world [7]. Many new *Bartonella* species have been described in recent years in conjunction with an expanding host range, and evidence supports transmission by a wide range of blood-sucking arthropod vectors that include ticks, sand flies, biting flies, fleas, and lice [8]. These bacteria are the causative agents of several diseases, including Cat Scratch disease (CSD) (*Bartonella henselae*), Carrion’s disease (*Bartonella bacilliformis*) and Trench fever (*Bartonella quintana*). In the U.S., cats are the main reservoir of *B. henselae*, humans and dogs are incidental hosts, and CSD is common with the highest *B. henselae* seroprevalence found in cats in warm and humid areas of the Southeast, Hawaii, coastal California, and the south central plains regions [9]. The cat flea (*Ctenocephalides felis* (Bouchè)) is the primary vector of *B. henselae*, but the pathogen can also be transmitted by cat bites and/or scratches contaminated with flea feces. Trench fever is also prevalent in the U.S. in homeless shelters; it is transmitted by the body louse (*Pediculus humanus humanus* L.), and humans are considered the main reservoir [10]. Based upon these patterns, detection of either *B. henselae* or *B. quintana* in bed bugs from the U.S. would seem more probable than other less prevalent *Bartonella* species that occasionally infect humans.

Post-resurgence efforts to screen bed bugs for human pathogens, including *Bartonella*, are few and relatively recent. Lowe and Romney [11] detected antibiotic resistant bacteria in five bed bugs collected from patients that were infected with the bacteria in Vancouver, British Columbia. They concluded that bed bugs can be vectors of methicillin-resistant *Staphylococcus aureus* and vancomycin resistant *Enterococcus faecium*. Richard et al. [12] screened 18 individual bed bugs from French warships for *Rickettsia* spp., *Bartonella* spp., and *Anaplasma* spp. These authors did not detect *Rickettsia* or *Bartonella* in the screened insects, but they found a single sample containing an Anaplasma-like bacterium “*Candidatus* Midichloria mitochondrii,” an endosymbiont of ticks [13]. To date, few studies have examined the vectorial capacity of bed bugs by screening wild populations for disease agents. Even fewer vector competency studies have been performed to implicate bed bugs as disease vectors.

*Bartonella* spp. have been detected in eastern bat bugs (*Cimex adjunctus* Barber) collected in two bat caves from the southeastern U.S. [14]. *Cimex adjunctus* is found in bat roosts, occasionally invading buildings in bat roosting sites [2]. It is a close relative of *C. lectularius* and co-infestation of human-built structures by both species can sometimes occur [2]. Because bats are relatively new hosts of *Bartonella* [15], and *C. lectularius* can occasionally feed on bats [2], [16], it is plausible that *C. lectularius* could also harbor bat-adapted *Bartonella* species. Given that both *C. lectularius* and several *Bartonella* species are resurgent in the U.S. in association with humans, bats, domestic pets, and wildlife, our objective was to investigate if the resurgence of bed bugs could represent a potential source of *Bartonella* transmission. We screened for *Bartonella* spp. in bed bug populations from geographic areas where *B. henselae* bacteremia is prevalent in feral and pet cat populations.

## Results and Discussion

**Figure 1:**
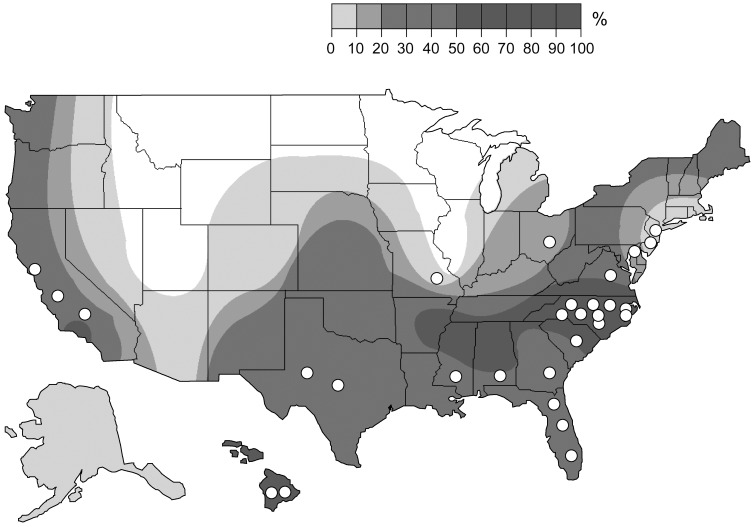
Distributions of collection sites and *B. henselae*. Bed bug collection sites (circles, *N* = 29) superimposed on a distribution map showing the percentage of pet cats with *B. henselae* antibodies from 33 geographic regions throughout the U.S. (adapted from Jameson et al. 1995 with permission from Oxford University Press). The highest seroprevalence of *B. henselae* is in warm, humid areas, especially in the southeastern U.S.

We did not detect any positive PCR products for *Bartonella* spp. in any of the 316 bed bugs freshly collected in the field between 2005 and 2010. Furthermore, we did not detect *Bartonella* spp. DNA in any of the bed bugs maintained in our cultures, including 10 bed bugs from two colonies maintained in the laboratory for 3 yr and five bed bugs from the Fort Dix (Harold Harlan) colony, which was originally collected in 1973, well before the recent resurgence. The endosymbiont *Wolbachia* is highly prevalent in bed bug populations [17], and we successfully amplified bacterial DNA from all 10 bed bugs screened with 16S rDNA universal primers. These results confirm that our negative PCR results for *Bartonella* are due to absence of *Bartonella* DNA and not PCR interference. Our findings suggest that bed bugs are an unlikely vector of *Bartonella* spp. However, as *Bartonella* are emerging pathogens, adaptation to new hosts in conjunction with an expanding range of vectors suggests that vector biologists should remain vigilant to the possibility of *Bartonella* occurring in bed bugs in the future [7]. The ecological interactions between *Bartonella* and bed bugs may be dynamic and could have changed since our collections were completed. We targeted our collections to regions of the United States where *B. henselae* seroprevalence is high in cats due to frequent flea exposure (Fig. 1). It is possible that other geographic regions, other *Bartonella* species, and other sites within the regions in which we collected might show different results. Vector competency studies, in which bed bugs are infected with the bacteria to determine the fate of the bacteria and the ability of bed bugs to infect a host, should be investigated to completely rule out bed bugs as potential vectors of *Bartonella* [18].

We screened 99 bed bugs from 10 apartments of an elderly housing building in Raleigh, North Carolina (Table 1). Five bed bugs from four different apartments yielded PCR products of the expected size for *Bartonella* spp. (400-600 bp), close to the *Bartonella henselae* control (604 bp). When these amplicons were sequenced, the alignments of the five sequences (Genebank accession numbers: KF286544-KF286548) with BlastX were 98% similar to a sequence of *Burkholderia multivorans,* accession number YP001949472.1. As these amplicons were generated using *Bartonella* 16S-23S intergenic spacer primers, it is possible that more specifically designed primers for *B. multivorans* may detect higher prevalence of this pathogen in bed bugs. *B. multivorans* is recognized as an important pathogen in nosocomial infections of patients with cystic fibrosis [19], although it has not been linked to an arthropod vector. Amplification and sequencing of *B. multivorans* DNA from multiple bed bugs in multiple but nearby apartments suggests that this association should be investigated further to determine whether bed bugs are competent to vector *B. multivorans* between humans.

**Table 1:** Sources of bed bugs screened by PCR for *Bartonella* spp.

State	City or county	Source	No. rooms collected	No. bed bugsPCR-screened	Year collected
Alabama	Mobile	Apartment	Single	10	2009
California	Los Angeles Co.	Hotel room	Single	10	2005
	San Rafael	House	Single	5	2009
	Whittier	Apartment	Single	9	2009
Florida	Broward Co.	Apartment	Single	9	2009
	Panama City	Hotel room	Single	10	2009
	Panama City	Hotel room	Single	8	2009
Georgia	Nocross	Hotel room	Single	10	2009
Hawaii	Honolulu	Apartment	Single	10	2009
	Honolulu	Apartment	Single	9	2009
Missouri	Springfield	Hotel room	Single	10	2009
New Jersey	Freehold	Apartment	Single	4	2005
North Carolina	Boone	Hospitality house	Multiple	4	2009
	Fayetteville	Homeless shelter	Multiple	9	2010
	Greenville	Apartment	Single	10	2009
	Raleigh^1^	Elderly housing	Single	9	2010
		Elderly housing^2^	Single	10	2010
		Elderly housing^2^	Single	10	2010
		Elderly housing	Single	10	2010
		Elderly housing	Single	10	2010
		Elderly housing	Single	10	2010
		Elderly housing	Single	10	2010
		Elderly housing^2^	Single	10	2010
		Elderly housing^3^	Single	10	2010
		Elderly housing	Single	10	2010
	Raleigh	Apartment	Multiple	9	2010
	Raleigh	Homeless shelter	Multiple	2	2010
	Wilmington	Homeless shelter	Multiple	3	2009
	Winston-Salem	Apartment	Single	10	2009
Ohio	Toledo	Apartment	Single	10	2007
South Carolina	Myrtle beach	Motel room	Single	10	2009
Texas	Beaumont	College dorm	Single	8	2009
	College Station	Apartment	Single	10	2008
Virginia	NA	Hospital	NA	8	2009
	Williamsburg	Apartment	Single	10	2009
Washington, DC	Washington, DC	Apartment	Multiple	10	2008
New Jersey	Fort Dix	Lab colony	NA	5	1973
New Jersey	NA	Lab colony	NA	5	2008
North Carolina	Winston-Salem	Lab colony	NA	5	2008

1 Bed bugs were collected from each of 10 apartments within a multi-unit elderly housing building.

2 One bed bug in this sample was positive for *Burkholderia multivorans.*

3 Two bed bugs in this sample were positive for *Burkholderia multivorans.*

NA = Information not available.

## Materials and Methods

### Sample Collection

Adult bed bugs were sampled from 36 unique collections in 29 different geographic locations spanning 13 states (Table 1, Fig. 1). A total of 331 bed bugs were screened individually for *Bartonella* spp. DNA, 2–10 bed bugs per collection (Table 1). Bed bugs were collected by pest control companies, collaborators, or by us and in all cases the resident or owner of the property gave permission to collect bed bugs from the site. In most locations specimens collected from a single room within an apartment or building were placed in a single collection vial, but in some samples bed bugs from multiple rooms were combined by the collector in a single vial. Most of the screened bed bugs (N = 316 bed bugs) were freshly field-collected and immediately stored in ethanol, but two collections were reared in the lab for approximately 3 yr before they were screened (N = 10 bed bugs). Additionally, we screened five bed bugs from the Fort Dix (Harold Harlan) colony which had been in culture since 1973, well before the bed bug resurgence in the late 1990s (Table 1).

### DNA Extraction and PCR Amplification

Bed bugs were surface sterilized by rinsing them with sterile water and 95% ethanol, and they were processed individually throughout the entire screening process. Total genomic DNA was extracted from individual adult bed bugs using the phenol-chloroform methodology described by Taggart et al. [20]. DNA concentration of samples was standardized to 20 ng/µL. *Bartonella* genus screening was performed using a polymerase chain reaction (PCR) assay targeting the 16S–23S ribosomal RNA intergenic transcribed spacer (ITS). The 16S–23S rRNA ITS region has been successfully used for the molecular diagnostic of *Bartonella* spp. from blood-sucking arthropods, including ticks and lice [21], [22]. For our screening, we used forward primer 438s (5′- GGTTTTCCGGTTTATCCCGGAGGGC-3′) and reverse primer 1100as (5′-GAACCGACGACCCCCTGCTTGCAAAGCA-3′) [23]. The detection limit observed in 100% of 10 replicate PCR reactions was 2.5 DNA copies of *B. henselae*. Bed bug DNA was spiked with 2.5 copies of *B. henselae* DNA to determine if PCR inhibitors would interfere with successful amplification. No PCR inhibitors were detected in bed bug DNA. Additionally, to ensure that we could amplify bacterial DNA from bed bug DNA samples, we randomly chose 10 bed bug DNA samples from 10 distinct geographic locations and amplified bacterial DNA using bacteria specific 16S rDNA universal primers: 27F (5′- AGAGTTTGATCMTGGCTCAG-3′) and 1492R (5′-TACGGYTACCTTGTTACGACTT-3′) [24]. Bacterial DNA was amplified from all 10 samples, but amplicons were not sequenced.

*Bartonella* spp. PCR amplification was performed in a 25 µL final volume reaction containing 12.5 µL of Premix Ex Taq™ (Takara Bio Inc.) and 7.5 µL of molecular grade water (Epicentre, Madison, WI). The reaction mixture was completed by adding 0.2 µL of forward and reverse primers (each at 30 µM) and 5 µL of either bed bug DNA template, positive control (with 2.5 copies of *Bartonella* DNA) or water (as PCR negative control). Amplification of the rRNA ITS region was performed under the following conditions: a hot start cycle of 94°C for 2 min, followed by 55 cycles of denaturing at 94°C for 15 s, annealing at 68°C for 15 s, and extension at 72°C for 18 s. Amplification was completed by an additional cycle of 72°C for 1 min to allow complete extension of PCR products. All amplification products were separated by electrophoresis in a 2% agarose gel and visualized under ultraviolet light with ethidium bromide. Positive and negative controls were used in all reactions and consisted of genomic DNA extracts of *B. henselae*, and molecular-grade sterile water, respectively. If a sample was found to be positive, the PCR reaction was purified using the QIAquick® PCR purification kit (QIAGEN, Valencia, CA) and sent to an external laboratory for sequencing. Alignment of sequences was performed with the program BlastX in order to identify bacteria at the genus and species levels.
